# Neo-Lymphoid Aggregates in the Adult Liver Can Initiate Potent Cell-Mediated Immunity

**DOI:** 10.1371/journal.pbio.1000109

**Published:** 2009-05-26

**Authors:** Melanie Greter, Janin Hofmann, Burkhard Becher

**Affiliations:** Division of Neuroimmunology, Institute of Experimental Immunology, Department of Pathology, University Hospital of Zurich, Zurich, Switzerland; National Jewish Medical and Research Center/Howard Hughes Medical Institute, United States of America

## Abstract

Subcutaneous immunization delivers antigen (Ag) to local Ag-presenting cells that subsequently migrate into draining lymph nodes (LNs). There, they initiate the activation and expansion of lymphocytes specific for their cognate Ag. In mammals, the structural environment of secondary lymphoid tissues (SLTs) is considered essential for the initiation of adaptive immunity. Nevertheless, cold-blooded vertebrates can initiate potent systemic immune responses even though they lack conventional SLTs. The emergence of lymph nodes provided mammals with drastically improved affinity maturation of B cells. Here, we combine the use of different strains of alymphoplastic mice and T cell migration mutants with an experimental paradigm in which the site of Ag delivery is distant from the site of priming and inflammation. We demonstrate that in mammals, SLTs serve primarily B cell priming and affinity maturation, whereas the induction of T cell-driven immune responses can occur outside of SLTs. We found that mice lacking conventional SLTs generate productive systemic CD4- as well as CD8-mediated responses, even under conditions in which draining LNs are considered compulsory for the initiation of adaptive immunity. We describe an alternative pathway for the induction of cell-mediated immunity (CMI), in which Ag-presenting cells sample Ag and migrate into the liver where they induce neo-lymphoid aggregates. These structures are insufficient to support antibody affinity maturation and class switching, but provide a novel surrogate environment for the initiation of CMI.

## Abstract 2

Are lymph nodes really essential for successful immunizations? We found that the liver can compensate for missing lymphoid structures in initiating cellular, but not antibody-mediated, immunity.

## Author Summary

Lymph nodes (LNs) are believed to be the most important tissues initiating immune responses by facilitating the activation of T and B lymphocytes. Mice lacking such LNs (called alymphoplastic) are severely immune compromised and resistant to immunizations. We discovered that the immune-deficiency of such alymphoplastic mice is actually not caused by the loss of LNs, but rather by the underlying genetic lesion. Surprisingly, mice lacking all lymph nodes can still mount potent T cell-mediated immune responses. We also discovered that T and B cells have completely different structural requirements for their activation/maturation. Whereas B cells rely on LNs to become efficient antibody-producing cells, T cells can be activated successfully outside of such dedicated tissues. So—in the absence of LNs—antigens delivered by immunization are actively transported into the liver where cellular immunity is initiated. The mammalian fetal liver is responsible for the early formation of blood and immune cells, and we propose that the adult liver can still provide a niche for T cell–antigen encounters. During evolution, T and B cells emerged simultaneously, allowing cold-blooded vertebrates (which lack LNs) to launch adaptive immune responses. The development of LNs in mammals coincided with a drastic improvement in antibody affinity maturation, whereas T cells remain LN-independent to this day.

## Introduction

Secondary lymphoid tissues (SLTs) are highly organized structures with defined compartments consisting of B and T cell areas. These distinct locations support the rapid circulation and concentration of Ag and the interaction of Ag-presenting cells (APCs) with lymphocytes. Prevailing dogma dictates that only if competent APCs transport Ag into SLTs, an adaptive immune response is initiated; otherwise, the Ag is ignored by the immune system [1]. For the initiation of humoral antibody (Ab)-mediated immunity in mammals, the formation of B cell follicles and germinal centers (GCs) appears to be a prerequisite. The dynamic nature of such GCs, including the interaction of follicular dendritic cells (FDCs) with B cells and Ag, was recently elegantly demonstrated by others [2]. However, in contrast to the B cell-dominated cortex, T cell areas, where T cells encounter mature APCs and their cognate Ag, are structurally ill defined. Whereas intravital confocal microscopy has provided compelling evidence for the capacity of SLTs to host T cell priming [3], definitive data supporting their absolute requirement for the initiation of T cell-mediated immunity (CMI) do not exist. In addition, cold-blooded vertebrates lacking conventional SLTs generate potent immune responses upon immunization. However, in the mammalian system, the apparent immunodeficiency of mice that lack SLTs strongly supports the notion that the initiation of effective immune responses requires the dedicated structures provided by SLTs [4]–[8]. *Alymphoplasia* (*aly/aly*) mice are characterized by a complete lack of lymph nodes (LNs) and Peyer's patches, and structural alterations of the spleen and thymus due to a point mutation in the NFκB-inducing kinase (NIK) [9]. NIK is vital for the initiation of the noncanonical NFκB cascade, which appears to play a discrete role, for instance, in the function of CD40 and lymphotoxin-β receptor (LTβR) signaling in some cell types [10]–[12]. *Aly/aly* mice display impaired Ab responses and loss of CMI, demonstrated by their inability to reject allogeneic grafts or tumors [4],[13],[14]. The developmental deficits in *aly/aly* mutants are readily explained by the requirement of NIK in LTβR signaling. LTβR is vital for the development of SLTs, and LTβR^−/−^ mice display similar developmental defects as do *aly/aly* mice or NIK^−/−^ mice [12],[15].

In this study, we describe that the immunodeficiency of *aly*/*aly* mice is not due to the absence of SLTs, but due to the impact of the underlying genetic defect on cellular immunity. Using different strains of alymphoplastic mice and T cell migration mutants in an experimental paradigm in which the site of Ag-delivery is distant from the site of priming and again distant from the site of inflammation, we can detect both T_H_ cell-driven autoimmune disease as well as systemic CTL-mediated antitumor immunity initiated through classical subcutaneous (s.c.) immunization/vaccination independent of SLTs. APCs present at the site of immunization migrate to and select the liver as a natural extra-lymphoid tissue for the initiation of CMI, which we propose to be an evolutionary hard-wired pathway already found in cold-blooded vertebrates. This alternative pathway, undescribed to this day, can potently drive CMI but fails to elicit B cell immunity, indicating that the immunization-induced T cell accumulation within conventional lymphoid organs mainly serves humoral immunity but that CMI can be initiated elsewhere.

## Results

### Autoimmunity Cannot Be Initiated in *Aly/Aly* Mice

**Figure 1:**
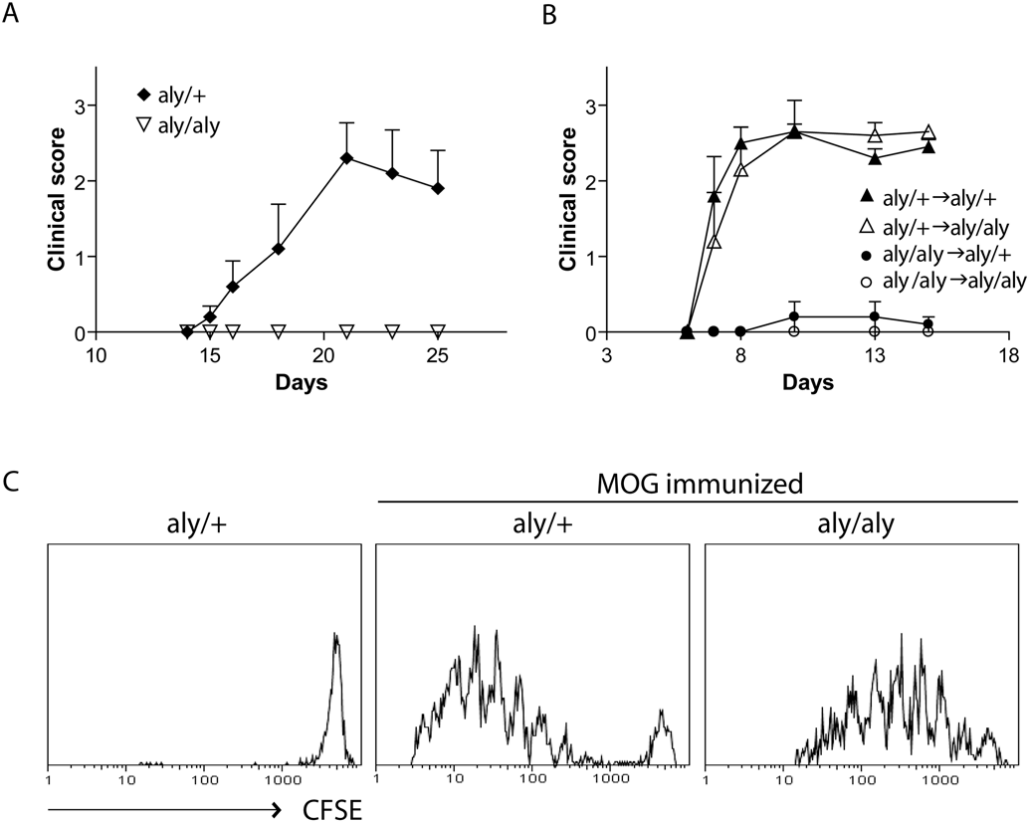
*Aly/aly* mice are resistant to the development of EAE. (A) EAE was induced by active immunization with MOG_35–55_/CFA in *aly*/*aly* (▿) and *aly*/+ (♦) mice. (B) EAE was induced by adoptive transfer of pathogenic T cells derived from MOG-immunized *aly/aly* or *aly*/+ donors into *aly*/*aly* or *aly*/+ recipients. *aly*/+ into *aly*/+: ▴, *aly*/+ into *aly*/*aly*: Δ, *aly/aly* into *aly*/+: •, and *aly/aly* into *aly*/*aly*: ○. Shown is a representative of two individual experiments (*n*≥5 mice/group)±SEM. (C) *Aly/+* and *aly*/*aly* mice were injected with 20×10^6^ CFSE-labeled splenocytes i.v. derived from 2D2 Tg mice and immunized s.c. with MOG_35–55_/CFA. At 4 dpi, splenocytes were analyzed by flow cytometry by gating on 2D2^+^ cells. Results are representative of two individual experiments (*n* = 2 mice/group).

We first sought to determine whether LNs are an absolute requirement for the induction of a complex T_H_ cell-driven autoimmune response initiated by the s.c. delivery of auto-Ag. Experimental autoimmune encephalomyelitis (EAE) is a B cell-independent, T_H_ cell-mediated demyelinating autoimmune disease of the central nervous system (CNS) and serves as the animal model for multiple sclerosis (MS). The conversion of T_H_ cells from the naive to effector state is vitally dependent on the structures provided by LNs [5],[6]. Cervical LNs are widely held to constitute the predominant intrinsic priming site for encephalitogenic T cells, based on the observation that these LNs support the expansion of PLP-TcR transgenic (Tg) T cells [16],[17]. However, draining inguinal LNs drive the polyclonal, endogenous T cell population after s.c. immunization with encephalitogenic peptides. To assess the role of SLTs in the transition of T_H_ cells from a naive to effector state (T cell priming), we induced EAE in *aly/aly* or *aly/+* mice by s.c. immunization with myelin oligodendrocyte glycoprotein peptide and complete Freud adjuvant (MOG_35–55_/CFA). Figure 1A shows that *aly/aly* mice are completely resistant to EAE compared to *aly/+* control mice (the latter developing normal SLTs as NIK is haplosufficient). To verify the notion that pathogenic T cells cannot be raised in *aly/aly* mice, they were immunized s.c. with MOG_35–55_. Eleven days postimmunization (dpi), splenocytes were harvested, and MOG_35–55_-reactive cells were expanded in vitro and subsequently transferred into *aly/aly* as well as *aly/+* recipients. Figure 1B shows that only cells derived from *aly/+* donors were able to induce disease regardless of whether the recipients had SLTs (*aly/+*) or not (*aly/aly*). In contrast, MOG_35–55_-reactive T cells derived from *aly/aly* donors were not pathogenic and did not mediate CNS inflammation.

To assess the capacity of LN-less mice to initiate T cell expansion in response to s.c.-delivered Ag, CFSE-labeled TcR Tg T cells (2D2) specific for the encephalitogenic MOG_35–55_ peptide [18] were adoptively transferred into either *aly/aly* or *aly/+* mice prior to immunization with their cognate Ag. After 4 d, splenocytes were analyzed for T cell expansion by flow cytometry (Figure 1C). Ag-specific T cell proliferation can be observed in *aly/aly* mice; however, they display slightly delayed kinetics in comparison to *aly/+* mice. Similar results were obtained with Ovalbumin (OVA) TcR Tg T cells (OTII) transferred into *aly/aly* and *aly/+* mice (unpublished data), indicating that T cell expansion can be initiated independent of SLTs, whereas efficient effector function is dependent on the microenvironment provided by SLTs.

### Induction of Productive T Cell Immunity in the Absence of SLTs

The fact that *aly/aly* mice do not develop T cell-driven autoimmune disease could be explained by their inability to prime self-reactive T cells (a) due to the lack of dedicated draining LNs [5],[6], or (b) due to a direct impact of the NIK mutation on immune cells [19],[20]. In order to define whether their EAE resistance is due to the lack of LNs or an intrinsic defect of *aly/aly* mice to prime T cells, we generated a series of bone marrow (BM)-chimeric mice. To restrict the NIK mutation to the hematopoietic system, lethally irradiated *aly/+* mice were injected with BM cells from *aly/aly* donor mice *(aly/aly*→*aly/+)*. Conversely, to conserve the developmental structural defects, without the NIK lesion of the hematopoietic compartment, *aly/aly* mice were reconstituted with BM cells of normal *aly/+* donors (*aly/+*→*aly/aly)*. As previously reported, spontaneous development of lymphoid tissues in *aly/aly* recipients upon reconstitution was expectedly not detected [21].

**Figure 2:**
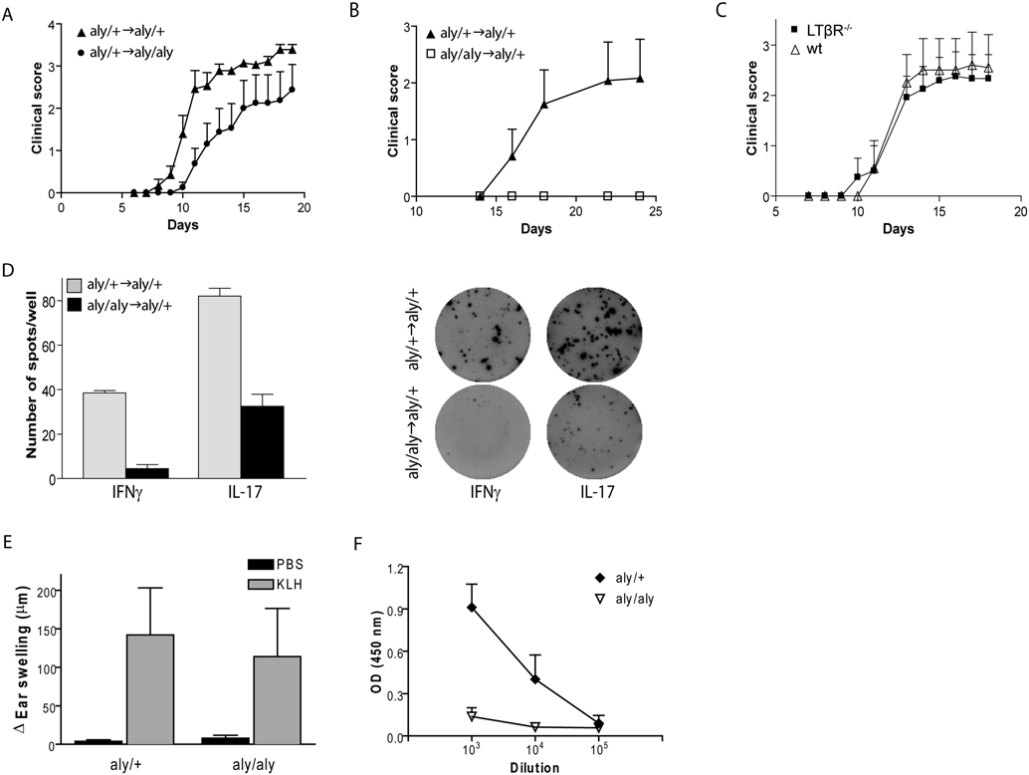
SLTs are crucial for B but not T cell-mediated immune responses. (A and B) EAE progression in BM-chimeras immunized s.c. with MOG_35–55_/CFA. (A) *aly*/+→*aly*/+: ▴, *aly*/+→*aly*/*aly*: •. (B) *aly*/*aly*→*aly*/+: □, *aly*/+→*aly*/+: ▴. (C) EAE was induced by active immunization with MOG_35–55_/CFA of LTβR^−/−^ mice (▪) and wt mice (Δ). Shown are representatives of three individual experiments (*n*≥5/group)±SEM. (D) LN-derived cells were obtained from *aly*/*aly*→*aly*/+ (black bars) and *aly*/+→*aly*/+ (grey bars) BM-chimeras 21 dpi with MOG_35–55_/CFA and rechallenged in vitro with 50 µg/ml MOG_35–55_ peptide to reveal IFNγ- and IL-17–secreting cells using Elispot. Shown is a representative of two individual experiments (*n* = 3/group)±standard deviation (SD). (E) DTH responses were induced by s.c. immunization with KLH/CFA of *aly/aly* and *aly/+* mice. At 11 dpi, the mice were challenged by intradermal injection of KLH (grey bars), or PBS (black bars) into the ear. Swelling was measured 24 h postchallenge using a precision caliper, and shown is the increase of ear swelling over baseline of a representative of three independent experiments (*n*≥2 mice/experiment)±SD. (F) Sera were collected from KLH-immunized *aly*/*aly* (▿) and *aly*/+ mice (♦) mice on 12 dpi and analyzed for the presence of total anti-KLH Abs by ELISA. Results are representative of three independent experiments (*n*≥2 mice/group)±SD.

Surprisingly, we discovered that *aly/+*→*aly/aly* BM-chimeras were fully susceptible to EAE after s.c. immunization with MOG_35–55_ (Figure 2A), clearly demonstrating that s.c. immunization can mount a productive T cell-driven autoimmune response even in the absence of draining LNs. Using the reciprocal approach, by generating *aly/+*→*aly/+* (WT-NIK immune system and normal SLTs) as well as *aly/aly*→*aly/+* BM-chimeras (NIK-deficient immune system and normal SLTs), we found that the NIK mutation lead to EAE resistance, even when the lymphoreticular compartment is unperturbed (Figure 2B). This finding clearly demonstrates that the reported immunodeficiency of *aly/aly* mice can largely be explained by the requirement of NIK for the initiation of immunity rather than the lack of LNs. In support of this, we found that unmanipulated LTβR^−/−^ mice, which also lack all LNs but have normal NIK function, are also fully susceptible to EAE (Figure 2C).

The formation of IFNγ and IL-17–secreting autoreactive T cells has been demonstrated to be a prerequisite for the development of autoimmunity [22]. In *aly/aly*→*aly*/+ mice we observed a substantial reduction in IL-17– and IFNγ-producing cells compared to the control mice *aly*/+→*aly*/+ (Figure 2D), indicating that the resistance to EAE in the absence of NIK could be related to the function of NIK in T cell polarization. The mechanistic underpinnings of this phenomenon are currently being investigated, but it is clear that the loss of NIK signaling impairs the capacity of *aly/aly* mice to generate pathogenic T_H_ cells regardless of their structural defects.

### B and T Cells Have Different Structural Requirements for Priming and Maturation

Given the dogma that in mammals, CMI initiated by s.c. or intramuscular Ag-delivery requires the presence of SLTs, it is feasible that the remaining SLT (i.e., the spleen) in *aly/+*→*aly/aly* BM-chimeras compensates for the absence of LNs. In order to test this notion, we splenectomized *aly/+*→*aly/aly* BM-chimeras (*aly/+*→*aly/aly* ^spl^) 14 d prior to the induction of EAE. Upon immunization, *aly/+*→*aly/aly* ^spl^ mice developed EAE with the same disease severity as control mice (Table 1). We noted a slight delay in disease onset when all SLTs are absent, while histopathological analysis of diseased mice revealed no difference between *aly/+*→*aly/+* and *aly/+*→*aly/aly* ^spl^ mice (Figure S1).

**Table 1:** Mice devoid of SLTs are fully susceptible to EAE.

BM-Chimeras	Incidence (%)	Mean Day of Disease Onset^a^	Mean Maximal Clinical Score^a^
*aly*/+→*aly*/+	94.7 (18 of 19)	11.1 (±0.4)	3.0 (±0.1)
*aly*/+→*aly*/+^spl^	75 (9 of 12)	11.8 (±0.2)	3.0 (±0.3)
*aly*/+→*aly*/*aly*	65.2 (15 of 23)	13.7 (±0.7)	3.3 (±0)
*aly*/+→*aly*/*aly* ^spl^	64.7 (11 of 17)	15.8 (±0.8)	3.4 (±0.1)
*aly*/*aly*→*aly*/+	0 (0 of 10)	—	—

a of diseased animals (±SEM).

**Figure 3:**
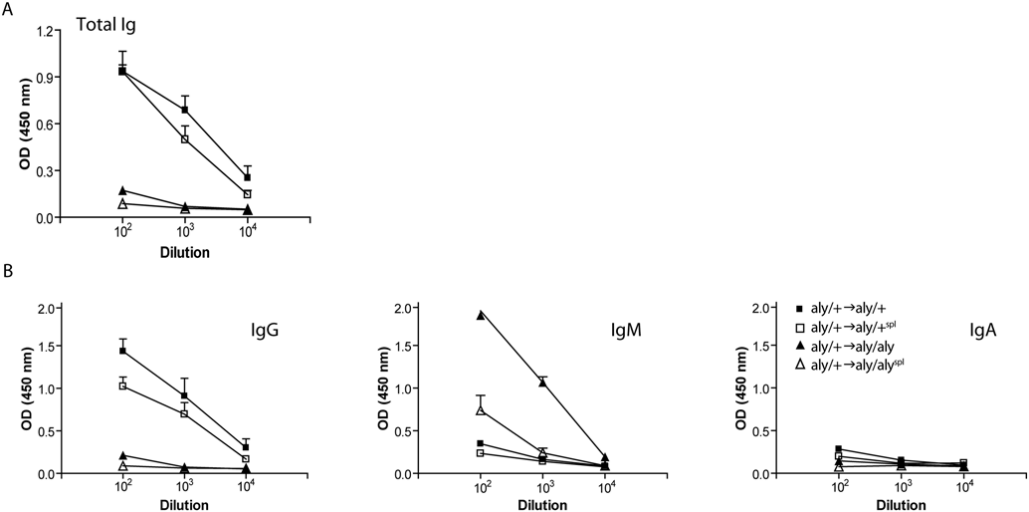
Ab response to s.c. auto-Ag depends on the presence of dedicated lymphoid structures. Titers of anti-MOG Abs (total Ig, IgG, IgM and IgA) determined from sera of diseased BM-chimeras immunized s.c. with MOG_35–55_/CFA by ELISA. *aly*/+→*aly*/+: ▪, *aly*/+→*aly*/+^spl^: □, *aly*/+→*aly*/*aly*: ▴, *aly*/+→*aly*/*aly* ^spl^: Δ. (A) shows total Ig, (B) shows IgG, IgM, and IgA. Shown is a representative of 3 individual experiments (*n* = 3/group)±SD.

In contrast to T cell activation, we found that B cell activation requires the structural environment provided by SLTs. To investigate the impact of immunization on T versus B cell responses, we used Keyhole limpet hemocyanin (KLH) as a model of foreign Ag to elicit delayed-type hypersensitivity (DTH) responses. *Aly/aly* as well as *aly/+* mice were immunized with KLH, and 11 dpi, they were challenged by intradermal injection with KLH into the ear. As illustrated in Figure 2E, both groups were able to mount a solid DTH reaction measured by ear swelling, which was only marginally lower in *aly/aly* than in *aly/+* mice. However, in contrast to ear swelling, which is indicative of CMI, *aly/aly* mice did not mount Abs against KLH when compared to *aly/+* mice, demonstrating that the development of a humoral immune response is ablated in the absence of lymphoreticular structures (Figure 2F). We could reproduce functional DTH responses using other Ags including OVA and MOG_35–55_ (unpublished data). Similarly, in our EAE paradigm using BM-chimeras, whereas control mice (*aly/+*→*aly/+* and *aly/+*→*aly/+* ^spl^) elicit high Ab titers, anti-MOG Abs are virtually absent in mice without LNs (either *aly/+*→*aly/aly* or *aly/+*→*aly/aly* ^spl^) (Figure 3A). Analysis of isotype subtypes revealed that in splenectomized alymphoplastic mice, elevated anti-MOG IgM could be detected, which has previously been reported [7],[23],[24], whereas class switching to IgG could not be observed (Figure 3B).

Taken together, and in agreement with the notion that SLTs are vital for B cell activation, highly organized SLTs are obligatory for the generation of high-affinity Igs and class switching, whereas potent cellular immunity can be induced successfully upon s.c. immunization even in the absence of SLTs.

### In the Absence of SLTs, Subcutaneously Delivered Ag Is Transported into the Liver

**Figure 4:**
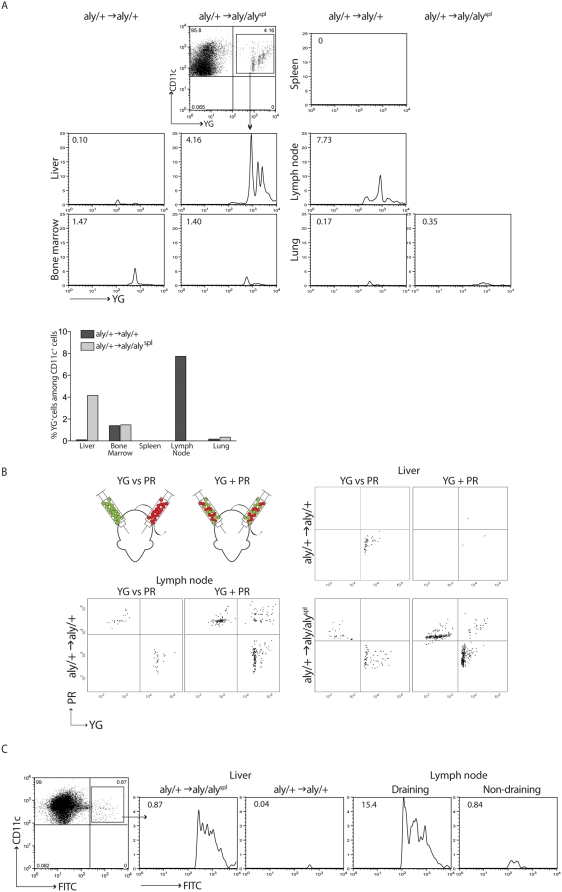
Ag-laden APCs migrate to the liver in the absence of SLTs. (A) *Aly* BM-chimeras were injected s.c. with YG microspheres/CFA, and various organs were analyzed by FACS for the presence of fluorescently labeled CD11c^+^ cells 7 dpi. Data represent one of three individual experiments. (B) *Aly* BM-chimeras were injected s.c. with either a mixture of YG and PR beads (YG+PR) into both flanks or YG beads into one flank and PR beads into the other flank (YG vs. PR). At 7 dpi, livers and LNs (only in *aly*/+→*aly*/+ mice) were analyzed by FACS for single (YG or PR) or double (YG and PR) positive APCs (gated on CD11c^+^ and CD11b^+^ cells). (C) *Aly* BM-chimeras were painted on the shaved flanks with 100 µl of 4 mg/ml FITC dissolved in 1:1 acetone:dibutylphalate. After 24 h, livers and, in *aly*/+→*aly*/+ mice, draining and nondraining inguinal LNs were analyzed by FACS for the presence of FITC^+^ cells (CD11c^+^).

Since the loss of SLTs in *aly* BM-chimeric mice does not hinder the development of T cell immunity, we wanted to determine at which alternative site T cell priming could take place and to which organ the Ag travels from the site of immunization (s.c.). Therefore, *aly* BM-chimeras were injected s.c. with yellow green (YG) carboxylate microspheres emulsified in CFA. At 7 dpi, various organs were isolated and analyzed for the presence of fluorescent cells by flow cytometry. Figure 4A shows that in control mice (*aly/+*→*aly/+*), fluorescently labeled APCs were exclusively detected in LNs upon s.c. immunization. It was previously shown that the BM has the capacity to drive an enriched population of high-affinity TcR Tg T cells in response to blood-borne Ag [25]. As expected, upon intravenous (i.v.) delivery of Ag, the vast majority of it accumulates in the spleen, BM, and liver, regardless of the presence of SLTs (Figure S2). However, after (s.c.) immunization of *aly/+*→*aly/aly* ^spl^ BM-chimeras lacking SLTs, APCs carrying fluorescent microspheres migrate primarily to the liver and not the other organs analyzed (thymus, CNS, and gut; unpublished data) (Figure 4A). Only a small amount of Ag reaches the liver when draining SLTs are present. Next, we wanted to determine the means of the Ag transport from the s.c. reservoir to the liver. To determine whether the Ag diffuses to the liver or is actively transported by APCs, *aly/+*→*aly/+* and *aly/+*→*aly/aly* ^spl^ chimeric mice were separated into two groups. One received YG microspheres/CFA in the left flank and polychromatic red (PR) microspheres/CFA in the right flank. The other group received a mixture of YG- and PR-coupled beads in both flanks (see scheme in Figure 4B). After 7 d, mice were sacrificed, perfused, and a single-cell suspension of livers, LNs, and spleens was generated for cytofluorometric analysis. We found that the mixture of PR/YG-coupled beads generated a large proportion of dual-labeled CD11b as well as CD11c-positive APCs. Conversely, the injection of either PR- or YG-coupled microspheres into each flank revealed merely single-labeled APCs in the liver. The presence of single-labeled cells within the liver strongly suggests that the Ag is delivered to the liver by the migration of APCs initially present at the site of immunization. Passive diffusion of the Ag from the site of immunization via the bloodstream to the liver cannot be fully excluded, but is evidently not the dominant means of Ag delivery. In addition, only a negligible amount of Ag reaches the liver when dedicated SLTs are present (Figure 4). We could also confirm these findings by using soluble FITC painted on shaved flanks (without the adjuvant CFA). Twenty-four hours after FITC skin painting, we found FITC^+^ APCs primarily in the liver, again supporting the notion that the liver can serve as an alternative Ag-presenting site when draining LNs are not available (Figure 4C).

### Extra-Lymphoid Aggregates in the Liver Host T Cell/APC Encounters

**Figure 5:**
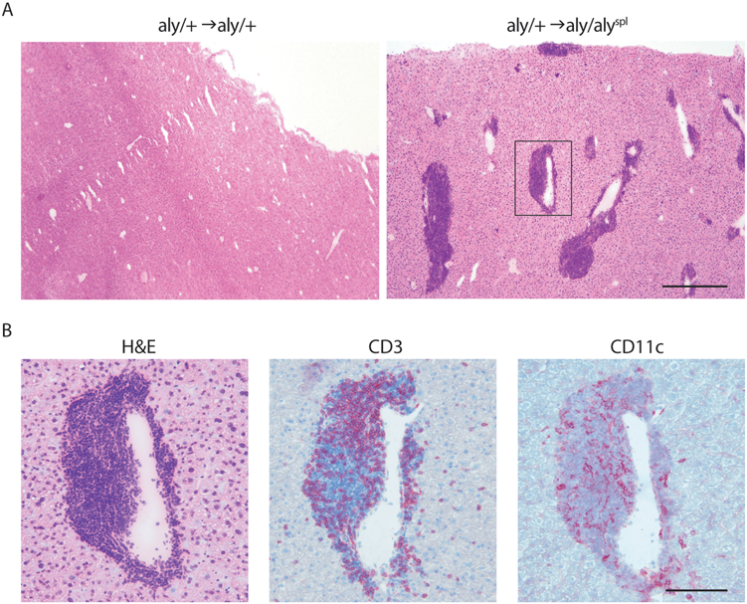
Extra-lymphoid aggregates in the liver host T cells and APCs. (A) Liver cryosections from *aly* BM-chimeras immunized s.c. with MOG_35–55_ (d7) were stained with H&E. Bar indicates 500 µm. (B) Higher magnification image of the region indicated by the square in (A) stained with H&E and mAbs against CD3 and CD11c. Bar indicates 100 µm.

In order to determine whether lymphoid-like structures can be found in the liver, we analyzed the livers of BM-chimeric mice immunized s.c. with MOG_35–55_/CFA by histology (7 dpi). Livers of *aly*/+→*aly/aly* ^spl^ BM-chimeras showed massive infiltration of leukocytes in comparison to *aly*/+→*aly*/+ control mice (Figure 5). Histological analysis displays dendritic cells (DCs) in close proximity to T cells in the infiltrated periportal areas of the liver, indicative of T cell priming by Ag-laden APCs (Figure 5B). In spite of the stroma's inability to respond to LTα/β, detailed histological analysis revealed the presence of VCAM and ICAM in the infiltrates as well as B cells (Figure S3) and even the presence of CXCL13 transcripts indicative of aggregates ability to recruit B cells (unpublished data). However, no evidence for GC formation could be obtained (Figure S3).

**Figure 6:**
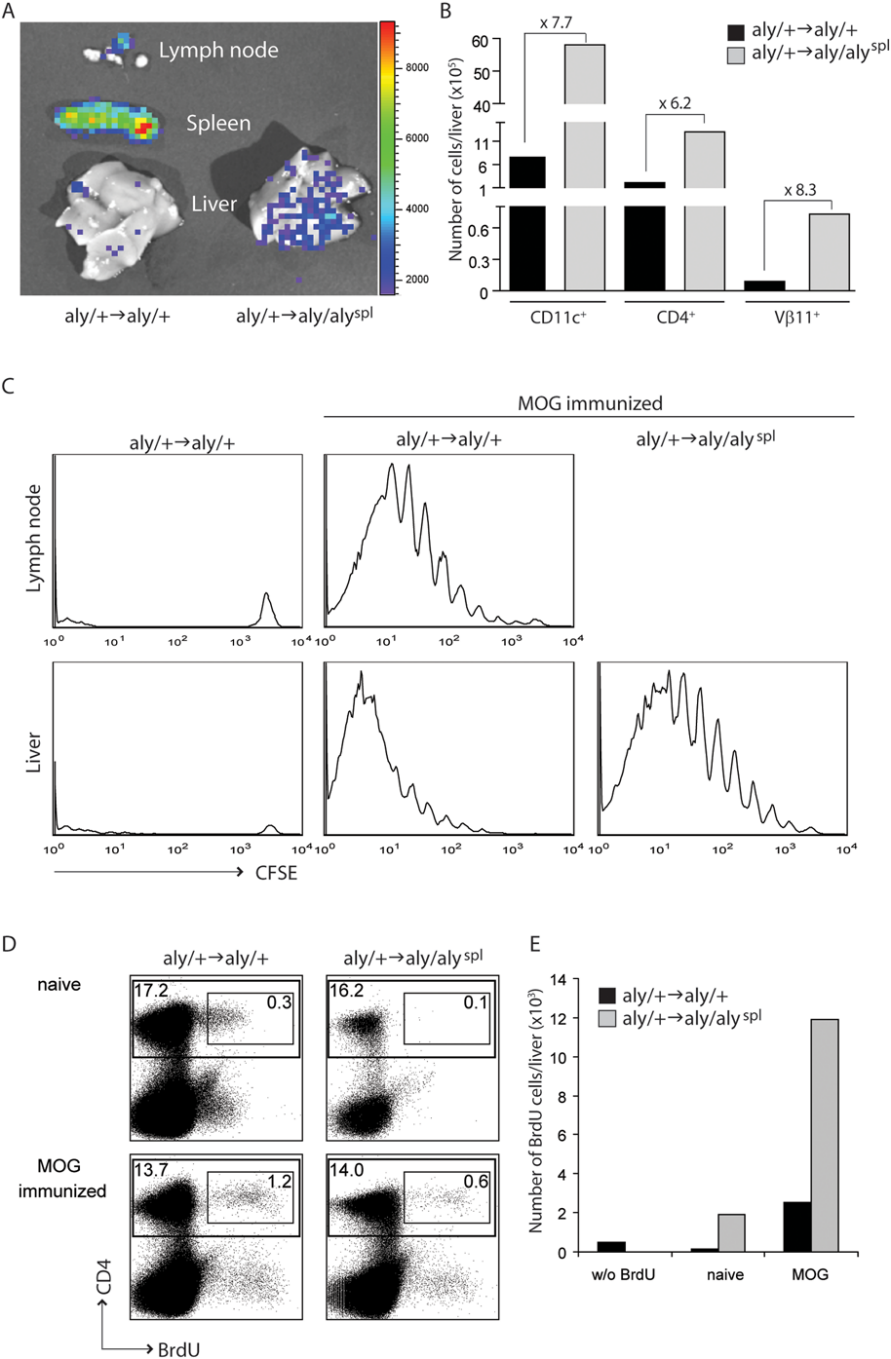
Accumulation and Ag-specific T cell expansion in the liver. *Aly* BM-chimeras were injected i.v. with 8×10^6^ Luc-2D2 Tg CD4^+^ T cells and immunized s.c. with MOG_35–55_/CFA. (A) At 2 dpi, mice were injected with luciferin, and after 10 min, sacrificed. Livers and, in control mice, LNs and spleen were isolated, and images were acquired by bioluminescence imaging to reveal the accumulation of the injected luciferase-positive (Luc-2D2) cells. (B) Absolute numbers of liver-invading DCs and Ag-specific T cells assessed from the percentage of CD11c^+^, CD4^+^, and Vβ11^+^ cells analyzed by flow cytometry. Numbers above the graph indicate the fold-increase of liver-invading cells of *aly/+*→*aly/aly* ^spl^ (grey) over *aly/+*→*aly/+* (black). (C) *Aly/+*→*aly/+* and *aly*/+→*aly/aly* ^spl^ BM-chimeras were injected with 8×10^6^ CFSE-labeled naive (CD62L^+^) CD4^+^ T cells derived from 2D2 Tg mice and immunized s.c. with MOG_35–55_/CFA. At 5 dpi, LNs (only in *aly/+*→*aly/+*) and liver-invading cells were analyzed by flow cytometry by gating on 2D2^+^ cells. (D and E) *Aly/+*→*aly/+* and *aly*/+→*aly/aly* ^spl^ BM-chimeras were immunized s.c. with MOG_35–55_/CFA. (D) At 7 dpi, BM-chimeras were injected with BrdU i.p. 30 min after BrdU injection, liver-invading cells were analyzed by flow cytometry for BrdU^+^ CD4^+^ cells. (E) Absolute numbers of liver-invading BrdU^+^ CD4^+^ T cells assessed by flow cytometry. *aly/+*→*aly/aly* ^spl^ (grey) and *aly/+*→*aly/+* (black).

We also transferred TcR Tg T cells from Luciferase-2D2 (Luc-2D2) mice into recipient BM-chimeras and observed the accumulation of Ag-responsive T cells in the liver 2 dpi with MOG_35–55_/CFA by bioluminescence imaging (Figure 6A). Figure 6B shows that the number of DCs (CD11c^+^) and adoptively transferred 2D2 T cells (CD4^+^/Vβ11^+^) is drastically increased in the liver in mice lacking SLTs.

In order to demonstrate that the observed lymphocyte accumulations in the liver can support cell expansion, we injected naive (CD62L^+^) CD4^+^ T cells derived from 2D2 Tg mice into *aly* BM-chimeras and subsequently immunized them with MOG_35–55_/CFA. At 5 dpi, livers were analyzed for Ag-specific CD4^+^ T cell proliferation. Even in normal mice, we find a large number of expanded T cells within the liver (Figure 6C), but one could argue that they have immigrated from their initial priming site, the draining LN. However, in the absence of SLTs, the livers of *aly/+*→*aly/aly* ^spl^ BM-chimeric mice are sufficient to propagate Ag-driven T cell expansion and accumulation. In order to confirm that Ag-specific T cell proliferation occurs in situ in the liver, we administered BrdU intraperitoneally (i.p.) into *aly* BM-chimeras 7 dpi with MOG_35–55_/CFA. Thirty minutes after BrdU injections, the mice were sacrificed, and livers were analyzed for proliferating (BrdU^+^) CD4^+^ T cells by flow cytometry. Figure 6D and 6E reveal the presence of BrdU^+^ cells in the livers of both *aly/+→aly/+* and *aly/+*→*aly/aly* ^spl^ BM-chimeras. The number of BrdU^+^ T cells in the liver is increased in *aly/+*→*aly/aly* ^spl^ BM-chimeras compared to the controls. The fact that we found such a rapid (30 min) emergence of proliferating T cells even in normal mice in which SLTs are present, indicates that some degree of liver-initiated CMI occurs simultaneously to the priming within draining LNs.

### The Adult Liver Can Support T Cell, But Not B Cell Priming

**Figure 7:**
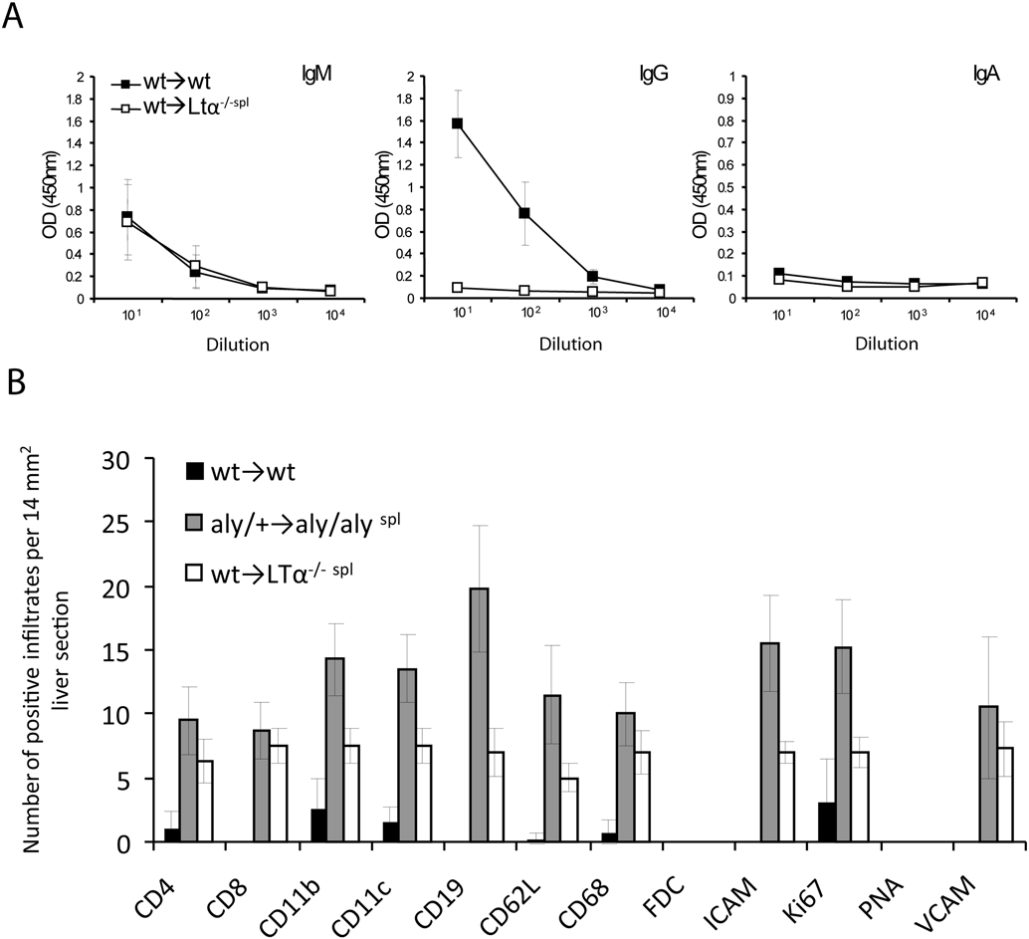
Surrogate liver aggregates support CMI, but not B cell maturation. Wt→wt and wt→LTα^−/− spl^ BM-chimeric mice were immunized s.c. with MOG_35–55_/CFA. (A) At 11 dpi, titers of anti-MOG Abs (IgG, IgM and IgA) were determined from sera by ELISA (*n* = 4 mice/group)±SD. (B) Liver sections from wt→wt, wt→LTα^−/−spl^, and *aly*/+→*aly/aly* ^spl^ BM-chimeras were stained with Abs against CD4, CD8, CD11b, CD11c, CD19, CD62L, CD68, FDC, ICAM, Ki67, PNA, and VCAM. Positively stained infiltrated areas of 14-mm^2^ liver sections were counted (*n* = 4 mice/group)±SD.

In contrast to our findings, which show that mice lacking SLTs do not generate high-affinity Ab-responses, intranasal influenza infection of splenectomized LTα^−/−^ mice reconstituted with wild-type (wt) stem cells, for instance, can initiate the formation of extra-lymphoid follicles within the lung, which support some degree of B cell maturation and Ab secretion [24],[26]. One possible explanation for these contrasting observations regarding Ab production is that in our case, stroma cells such as FDCs cannot signal through LTβR due to the mutation within NIK and that this could be the reason for our inability to observe GC formation and Ab secretion, whereas Moyron-Quiroz et al. [24],[26] used mice in which the stroma compartment can be engaged by LTα/β. To definitively address whether the stroma's inability to signal through NIK is the reason for the weak B cell response, we obtained LN-deficient LTα^−/−^ mice and reconstituted their hematapoietic system with wt stem cells. The resulting chimeras were splenectomized and lacked all peripheral SLTs (analogous to the *aly*/+→*aly*/*aly* ^spl^). Yet in contrast to *aly*/+→*aly*/*aly* ^spl^, wt→LTα^−/−spl^ chimeras have normal stromal cell function, and FDCs are capable of responding to LTα/β. These mice were immunized s.c., and the formation of B cell maturation and Ab production was analyzed. Figure 7A demonstrates that these wt→LTα^−/−spl^ chimeras behave exactly like *aly*/+→*aly*/*aly* ^spl^ in regards to their inability to generate high Ab titers and to class switch.

In a comparative fashion, we analyzed the histological parameters of wt→wt, *aly*/+→*aly*/*aly* ^spl^, and wt→LTα^−/−spl^ chimeras (Figure 7B). Although only alymphoplastic mutants revealed the presence of lymphoid aggregates surrounding periportal areas of the liver, neither FDCs nor PNA-positive clusters could be found, again supporting the notion that the surrogate structures in the liver support T cell function but fail to initiate the formation of GCs needed for Ab-affinity cell maturation and class switching. Lastly, the large number of Ki67^+^ cells within the liver aggregates again support our conclusion, that active proliferation within the liver can be induced by s.c. immunization (Figure 7B).

### Priming of Cytotoxic Antitumor T Cells Independent of SLTs

**Figure 8:**
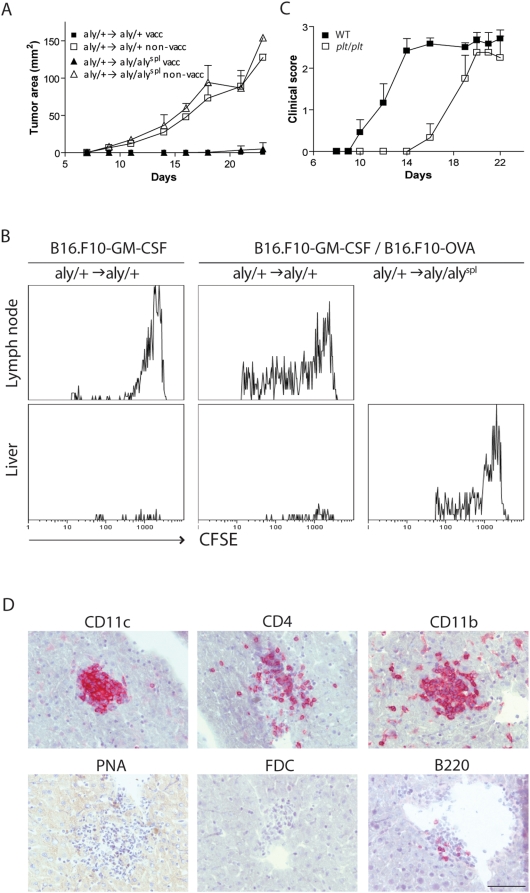
CD8^+^ T cell priming in the liver and lymphoid aggregates in *plt/plt* mice. (A) Tumor progression of *aly* BM-chimeras. Mice were vaccinated s.c. with 1 ×10^6^ irradiated GM-CSF-B16.F10 cells into one flank and 12 d later, mice received 2×10^5^ live B16.F10-Luc cells into the opposite flank. Vaccinated *aly*/+→*aly*/+: ▪; nonvaccinated *aly*/+→*aly*/+: □; vaccinated *aly*/+→*aly*/*aly* ^spl^: ▴; and nonvaccinated *aly*/+→*aly*/*aly* ^spl^: Δ. (B) *aly/+*→*aly/+* and *aly*/+→*aly/aly* ^spl^ BM-chimeras were injected i.v. with 20×10^6^ CFSE-labeled splenocytes from OTI Tg mice and s.c. injected with a mix of 1×10^6^ B16.F10-OVA and 1×10^6^ B16.F10-GM-CSF cells. At 12 dpi, LNs (only in *aly/+*→*aly/+*) and liver-invading cells were analyzed by flow cytometry for the proliferation of CD8^+^ OTI cells (Vα2^+^). (C) EAE progression of *plt/plt* (□) and wt (▪) mice immunized s.c. with MOG_35–55_/CFA. (D) Liver cryosections from diseased *plt/plt* mice (C) were stained with mAbs against CD11c, CD11b, CD4, FDC, B220, and PNA. Bar indicates 100 µm.

Although we have demonstrated the development of T_H_ cell-driven autoimmune disease in mice lacking SLTs, we wanted to elucidate whether these mice are also capable of inducing successful CTL immunity. We used the B16.F10 murine melanoma model, which represents a lethal and poorly immunogenic cancer. Irradiated GM-CSF expressing B16.F10 cells are used as s.c. vaccine to initiate potent CD8^+^-antitumor immunity against live parental B16.F10 tumor cells [27]. We injected irradiated B16.F10-GM-CSF cells s.c. into one flank of *aly/+*→*aly/+* and *aly/+*→*aly/aly* ^spl^ chimeric mice. At 12 dpi, mice were challenged with parental B16.F10 cells injected into the opposite flank. Figure 8A shows that *aly/+*→*aly/aly* ^spl^ chimeric mice can elicit potent antitumor CTL responses revealed by the inhibition of tumor growth. Next, we transferred CFSE-labeled MHC class I-restricted OVA-TcR Tg OTI T cells into *aly/+*→*aly/aly* ^spl^ and *aly/+*→*aly/+* BM-chimeric mice and subsequently injected irradiated B16.F10 cells expressing OVA. At 12 dpi, livers and, in control animals, also spleen and LNs were analyzed by FACS for Ag-specific CD8^+^ T cell expansion. As demonstrated in Figure 8B, proliferation of CD8^+^ OTI cells was detected in the liver of mice lacking SLTs. Hence, even under conditions in which the draining LNs are considered a compulsory site hosting the encounter of captured Ag and infiltrating CD8^+^ T cells, we can detect potent T cell responses, which originate in the liver when SLTs are absent.

### Liver Follicles Are Induced by Immunization and Aberrant Homeostatic T Cell Migration

We next wanted to address the relevance of the liver to serve as an alternative priming site in a setting where LNs are present but T cell migration into LNs is defective. To this end, we analyzed *plt/plt* (paucity of LN T cells) mice, which display undisturbed B cell zones but severely abrogated T cell zones due to the loss of CCL19 and CCL21, which results in the inhibition of both naive T cell and DC homing into SLTs [28]. We found that *plt/plt* mice also developed delayed but fulminant EAE after s.c. immunization with MOG_35–55_/CFA (Figure 8C). Examination of liver sections of immunized *plt/plt* mice again revealed lymphocyte aggregates consisting mainly of CD4^+^ T cells and DCs within the liver (Figure 8D).

## Discussion

S.c. immunization instigates a situation in which draining LNs are widely held to be absolutely obligatory for the initiation of adaptive immunity. In the absence of such draining LNs, we found however, that APCs take up the Ag at the site of immunization and subsequently select the liver as an extra-lymphoid environment for the initiation of CMI. These findings are consistent with the propensity of alymphoplastic mice (NIK^−/−^, LTα^−/−^, and LTβR*^−/^* ^−^) to develop abnormal lymphocytic infiltrates primarily in the liver [15],[29]. The lymphocyte accumulation seen in the liver of naive alymphoplastic mice does not coincide with any overt tissue damage, nor do they develop any secondary sign of hepatic injury (M. Heikenwaelder, Zurich, Switzerland, personal correspondence). Such surrogate structures are evidently not as sophisticated as true SLTs and fail to support B cell priming, but are clearly sufficient to support CMI. Such neo-lymphoid structures in the liver are not restricted to alymphoplastic mouse strains, but can be reproduced in mice in which T cells do not migrate into the LNs (*plt/plt*). The fact that we observe the rapid emergence of immunization-induced T cell expansion in the liver of normal mice supports the notion that the adult liver provides an efficient niche for the initiation of CMI. Moyron-Quiroz et al. [26] elegantly demonstrated that the lymphoid tissue in the lung (BALT) is sufficient to generate immunity against an infectious agent attacking the lung. In their experimental paradigm, peripheral SLTs are not compulsory for the initiation of protective immunity, and they could even observe some degree of B cell maturation. In our report, however, after s.c. immunization, the local APCs must sample the Ag and then actively migrate to and select the liver as a site for T cell priming, which then is even capable of driving autoimmune responses within the CNS. In our experimental paradigm, the site of Ag deposition, priming, and inflammation are distinct. The liver is thus not like the BALT or the NALT, a site where local immune responses can be initiated, but represents a niche for systemic T cell priming under conditions in which the draining LNs are widely held to be absolutely compulsory. The fact that Ag-laden APCs migrate from the site of immunization to the periportal areas in the liver could be explained by the presence of chemoattractive factors in the liver aggregates observed in SLT mutants. Alternatively, the extensive lymphatic network of the liver makes it an ideal niche for the accumulation of leukocytes as a reservoir when regular SLTs are inaccessible.

Although the induction of CMI is not a function traditionally attributed to the adult liver, the fetal liver is a primary lymphoid organ hosting early hematopoiesis. Our findings suggest that the liver has the potential to “remember” its lymphoid function. The phenomenon, that, for instance, food allergies can be transferred by the transplantation of livers from an allergic donor to a previously nonallergic recipient [30], can be explained by our findings. Such transplant-acquired food allergy has only been described for the liver and not for other transplanted organs of the same donor [30]. It has been hypothesized that this occurrence is due to donor-derived allergen-specific lymphocytes residing in the liver. In support of this, Klein and Crispe [31] reported recently that after liver transplantation in a mouse in which Ag presentation was restricted to resident cells of the liver grafts, efficient CD8^+^ T cell priming can be induced locally in the transplanted liver.

The situation also is reminiscent of the effect of immunizations on some cold-blooded vertebrates that are much more primitive than mammals in their SLT organization (i.e., lacking GCs and showing only minimal affinity maturation). Frog tadpoles (*Alytes obstetricans*) immunized with rabbit serum in CFA developed a large accumulation of lymphocytes in the liver visible 2–3 wk after injection (L. Dupasquier, Basel, Switzerland, personal correspondence). Interestingly, during evolution, the emergence of RAG was permissive for the development of adaptive immunity in jawed fish [32]. RAG mediates somatic recombination and is required for the formation of both B and T cell receptors, which appear to have emerged simultaneously during evolution. However, whereas the adaptive immune system is well developed in the oldest jawed vertebrates (cartilaginous fish, e.g., sharks), potent affinity maturation, Ig-class switching, and GC formation are lacking. Class switching only appeared at the time of the divergence of amphibians [33]. The fact that CMI evolved earlier than modern humoral immune responses corroborates our discovery that T cells can function outside of dedicated lymphoreticular structures.

In summary, we demonstrate that the structural requirements for the initiation of B and T cell responses differ significantly. We found that B cells are dependent on the topography of dedicated lymphoid tissues, whereas CD4^+^ as well as CD8^+^ T lymphocytes retain the capacity to recognize Ag in a structure-independent fashion. This finding has obvious implications for our understanding of adaptive immunity and vaccination. As for the development of autoimmune diseases, our findings show that self-reactive T cells may not need to be primed in tissue-draining LNs, but could occur at the inflammatory site or even in organs distant to the target tissue.

## Materials and Methods

### Mice

C57BL/6 mice were purchased from Janvier Laboratories. Alymphoplasia (*aly/aly*) mice were obtained from Clea Laboratories and bred in-house under specific pathogen-free (SPF) conditions. Heterozygous *aly* (*aly*/+) mice were used as controls for homozygous *aly* mice (*aly*/*aly*); 2D2 (MOG-TCR Tg) mice were provided by V. Kuchroo (Harvard Medical School, Boston, Massachusetts); LTβR^−/−^ and LTα^−/−^ mice were provided by A. Aguzzi and M. Heikenwalder (University Hospital Zurich, Zurich, Switzerland); and OTII and OTI mice were purchased from Jackson Laboratories. Luciferase (pbActin-Luciferase) Tg mice were obtained from C. Contag (UCSF) and crossed to the 2D2 mice (Luc-2D2). *Plt/plt* mice were obtained from B. Ludewig (Kantonsspital St. Gallen, Switzerland). All mice were bred in-house under SPF conditions. BM-chimeras were generated as described previously [34]. Mice were splenectomized as described previously [35]. Animal experiments were approved by the Swiss veterinary Office (68/2003, 70/2003, 10/2006, and 13/2006).

### Induction of EAE

MOG_35–55_ peptide (MEVGWYRSPFSRVVHLYRNGK) was obtained from GenScript. EAE was induced as described previously [34] with the modification that BM-chimeras were generally not boosted with pertussis toxin. For adoptive transfer, MOG-reactive lymphocytes were generated as described [34]. Each time point shown is the average disease score of each group±the standard error of the mean (SEM).

### Leukocyte Isolation

Mice were euthanized with CO_2_, and various organs were removed to isolate leukocytes: For isolating lung cells, lungs were incubated with DNase (0.5 mg)/Liberase (1 mg/ml) (Roche) for 30 min at 37°C. Spleen, LNs, thymus, and lung were homogenized, and BM cells were isolated by flushing the bones with PBS. Cells were strained through a 100-µm nylon filter (Fisher) and washed. Erythrocytes of whole blood, BM, and spleen were lysed. For isolating hepatic nonparenchymal cells, the liver was incubated with DNase/Liberase for 30 min at 37°C, homogenized, and then centrifuged at room temperature (RT) for 2 min at 50*g*. The supernatant was then centrifuged at 1,500 rpm for 10 min, and the pellet was resuspended in 30% Percoll (Pharmacia) and centrifuged at 12,000 rpm for 30 min at 4°C. The interphase cells were collected and washed. For isolating intestinal lymphocytes, intestines were opened longitudinally, washed, and then cut into small pieces. Tissues were then incubated with DNase/Liberase and leukocytes were isolated using a percoll gradient as described above. Isolation of CNS lymphocytes has been described previously [35].

### Proliferation Assay

Mice were injected i.v. with 20×10^6^ CFSE (carbofluorescein diacetate succinimidyl ester)-labeled (Invitrogen/Molecular Probes) (10 µM) splenocytes obtained from either 2D2, OT-II, or OT-I TcR Tg mice or with 8×10^6^ CFSE-labeled naive CD4^+^ 2D2 Tg T cells (isolated with CD4^+^CD62L^+^ isolation kit from Miltenyi). Mice were subsequently immunized s.c. with 200 µg of MOG_35–55_./CFA (Adjuvant complete H37 Ra..; DIFCO) (for 2D2), OVA_323–339_/CFA (for OT-II), or with a 1∶1 mix of irradiated 2×10^6^ B16.F10-GM-CSF/B16.F10-OVA cells (for OT-I). At 4 or 5 dpi (12 dpi for OT-I), mice were sacrificed, and spleen, LNs (if present), and livers were analyzed by fluorescence-activated cell sorting (FACS) for the proliferation of CD4^+^ T cells using the clonotypic TcR and CFSE fluorescence (2D2: TCR Vα3.2 Ab; OT-II and OT1: Vα2 Ab).

### Histology and Flow Cytometry

Tissues were freshly snap-frozen in liquid nitrogen. To determine infiltration of inflammatory cells, tissue sections were stained with hematoxylin and eosin (H&E) or with the following mouse-specific Abs as previously described [34]: anti-CD11c (Jackson ImmunoResearch Labs), anti-CD11b (BMA Biomedicals), anti-CD3, anti-CD4, anti-CD19, anti-FDC M1, and anti-Thy1.1 (BD-Pharmingen), anti-ICAM, anti-VCAM, and anti-CD8 (Serotec). GC cells were stained with peanut agglutinin (PNA; Vector Laboratories).

For FACS analysis, the following Abs were used: anti-CD11c, anti-CD4, anti-CD8, anti-CD11b, anti-Vα3.2, anti-Vα3, and anti-Thy1.1 (BD-Pharmingen). The cells were analyzed using a FACS-Canto (BD) with Cell-Diva software. Postacquisition analysis was performed using FLOWJO software. To trace the distribution of Ag after immunization, mice were injected s.c. with 200 µl of yellow-green (YG) or polychromatic red (PR) 1.0-µm microspheres (Polysciences) emulsified in CFA. At 7 dpi, mice were euthanized with CO_2_, and organs were removed to isolate lymphocytes as described above. Single-cell suspensions were analyzed by FACS for the presence of fluorescein isothiocyanate (FITC^+^) or PE^+^ cells.

For FITC skin painting, mice were painted on the shaved flanks with 100 µl of 5 mg/ml FITC (Molecular Probes) dissolved in 1∶1 acetone:dibutylphtalate. On day 1, mice were euthanized with CO_2_, and organs removed and analyzed by FACS as described above.

### Delayed-Type Hypersensitivity (DTH) Assay

Mice were immunized s.c. with 100 µg/flank of KLH (Sigma) emulsified in CFA. At 11 dpi, mice were challenged by injecting 10 µg/10 µl KLH, PBS into the dorsal surface of the ear. DTH responses were determined by measuring the ear thickness using a caliper micrometer (Mitutoyo) 24 h after challenge, and Δ ear swelling was established by the increase in ear thickness over baseline (prechallenge ear thickness).

### Enzyme-Linked Immunosorbent Assay (ELISA)

Plates were coated with 10 µg of rMOG_1–121_ in 0.1 M NaHCO_3_ (pH 9.6) at 4°C overnight or KLH (Sigma), and blocked with 1% (w/v) bovine serum albumin (BSA). Diluted sera were incubated for 2 h at RT. After washing, peroxidase-conjugated antibodies to mouse immunoglobulins, IgG, IgA, and IgM (Sigma) were added (1∶1,000 diluted) and incubated for 1 h at RT. Plates were washed, and chromogen (Biosource) was added. Absorbance was measured on a microplate reader (450 nm) (Bio-Rad).

### Enzyme-Linked Immunospot Analysis (Elispot)

A total of 2×10^5^ cells were plated in medium containing 10% FCS and 50 µg/ml of MOG_35–55_ in 96-well plates (Millipore) coated with the capture Ab against either IFNγ or IL-17A [36]. Elispots were revealed as described previously [36] and subsequently analyzed on an Elispot reader (CTL immunospot).

### Bioluminescence Imaging

To visualize Luc-2D2 cells, mice were injected i.p. with 3 mg of luciferin (Xenogen) prior to bioluminescence imaging using an IVIS100 imaging station (Xenogen). The luminescent image was overlaid on the photographic image.

### Bromodeoxyuridine (BrdU) Treatment

Mice were immunized s.c. with MOG_35–55_/CFA. At 7 dpi, BrdU (BD Pharmingen) (2.5 mg) was injected i.p. 30 min before the mice were sacrificed and analyzed for proliferating (BrdU^+^) CD4^+^ T cells by flow cytometry with anti-BrdU Ab (eBioscience).

### Tumor Induction

Mice were s.c. vaccinated into one flank with irradiated (6,000 rads) 1×10^6^ B16.F10-GM-CSF cells. At day 12 after vaccination, mice were injected with live 2×10^5^ B16.F10-Luc cells into the opposite flank. Each time point shown is the average tumor size of each group±SEM, measured using a caliper.

## Supporting Information

Figure S1 Inflammatory lesions in the CNS of mice lacking SLTs. H&E stainings of spinal cord sections of diseased *aly*/+→*aly*/+ and *aly*/+→*aly*/*aly* ^spl^ BM-chimeras. Lower row represents higher magnification of the insert in upper row. Bar in upper row indicates 200 µm and in lower row 50 µm.
(3.79 MB TIF)

Figure S2 Intravenously delivered Ag accumulates in the spleen, BM, and liver. *Aly* BM-chimeras were injected i.v. with YG microspheres, and various organs were analyzed by FACS for the presence of fluorescently labeled APCs 7 dpi. Data represent one of three individual experiments.
(0.63 MB TIF)

Figure S3 Expression of lymphoid structure markers in livers of *aly BM*-chimeric mice. Liver cryosections from *aly* BM-chimeras immunized s.c. with MOG_35–55_ (d11) were stained with antibodies against CD4, CD8, CD11b, CD11c, CD19, CD62L, CD68, FDC, ICAM, Ki67, PNA, and VCAM. Bar indicates 200 µm.
(17.11 MB TIF)

## Abbreviations

Ab: antibody
APC: Ag-presenting cell
BM: bone marrow
CMI: cell-mediated immunity
CNS: central nervous system
DC: dendritic cell
dpi: days postimmunization
EAE: experimental autoimmune encephalomyelitis
FDC: follicular dendritic cell
GC: germinal center
i.p.: intraperitoneally
LN: lymph node
PR: polychromatic red
s.c.: subcutaneous
SEM: standard error of the mean
SLT: secondary lymphoid tissue
Tg: transgenic
wt: wild-type
YG: yellow green

## References

[1] Zinkernagel RM, Ehl S, Aichele P, Oehen S, Kundig T (1997). Antigen localisation regulates immune responses in a dose- and time-dependent fashion: a geographical view of immune reactivity.. Immunol Rev.

[2] Schwickert TA, Lindquist RL, Shakhar G, Livshits G, Skokos D (2007). In vivo imaging of germinal centres reveals a dynamic open structure.. Nature.

[3] Beltman JB, Maree AF, Lynch JN, Miller MJ, de Boer RJ (2007). Lymph node topology dictates T cell migration behavior.. J Exp Med.

[4] Ochsenbein AF, Sierro S, Odermatt B, Pericin M, Karrer U (2001). Roles of tumour localization, second signals and cross priming in cytotoxic T-cell induction.. Nature.

[5] Karrer U, Althage A, Odermatt B, Roberts CW, Korsmeyer SJ (1997). On the key role of secondary lymphoid organs in antiviral immune responses studied in alymphoplastic (aly/aly) and spleenless (Hox11(-/-)) mutant mice.. J Exp Med.

[6] Rennert PD, Hochman PS, Flavell RA, Chaplin DD, Jayaraman S (2001). Essential role of lymph nodes in contact hypersensitivity revealed in lymphotoxin-alpha-deficient mice.. J Exp Med.

[7] Fu YX, Huang G, Wang Y, Chaplin DD (2000). Lymphotoxin-alpha-dependent spleen microenvironment supports the generation of memory B cells and is required for their subsequent antigen-induced activation.. J Immunol.

[8] Matsumoto M, Mariathasan S, Nahm MH, Baranyay F, Peschon JJ (1996). Role of lymphotoxin and the type I TNF receptor in the formation of germinal centers.. Science.

[9] Shinkura R, Kitada K, Matsuda F, Tashiro K, Ikuta K (1999). Alymphoplasia is caused by a point mutation in the mouse gene encoding Nf-kappa b-inducing kinase.. Nat Genet.

[10] Senftleben U, Cao Y, Xiao G, Greten FR, Krahn G (2001). Activation by IKKalpha of a second, evolutionary conserved, NF-kappa B signaling pathway.. Science.

[11] Garceau N, Kosaka Y, Masters S, Hambor J, Shinkura R (2000). Lineage-restricted function of nuclear factor kappaB-inducing kinase (NIK) in transducing signals via CD40.. J Exp Med.

[12] Yin L, Wu L, Wesche H, Arthur CD, White JM (2001). Defective lymphotoxin-beta receptor-induced NF-kappaB transcriptional activity in NIK-deficient mice.. Science.

[13] Shinkura R, Matsuda F, Sakiyama T, Tsubata T, Hiai H (1996). Defects of somatic hypermutation and class switching in alymphoplasia (aly) mutant mice.. Int Immunol.

[14] Lakkis FG, Arakelov A, Konieczny BT, Inoue Y (2000). Immunologic ‘ignorance’ of vascularized organ transplants in the absence of secondary lymphoid tissue.. Nat Med.

[15] Futterer A, Mink K, Luz A, Kosco-Vilbois MH, Pfeffer K (1998). The lymphotoxin beta receptor controls organogenesis and affinity maturation in peripheral lymphoid tissues.. Immunity.

[16] de Vos AF, van Meurs M, Brok HP, Boven LA, Hintzen RQ (2002). Transfer of central nervous system autoantigens and presentation in secondary lymphoid organs.. J Immunol.

[17] Zhang H, Podojil JR, Luo X, Miller SD (2008). Intrinsic and induced regulation of the age-associated onset of spontaneous experimental autoimmune encephalomyelitis.. J Immunol.

[18] Bettelli E, Pagany M, Weiner HL, Linington C, Sobel RA (2003). Myelin oligodendrocyte glycoprotein-specific T cell receptor transgenic mice develop spontaneous autoimmune optic neuritis.. J Exp Med.

[19] Ishimaru N, Kishimoto H, Hayashi Y, Sprent J (2006). Regulation of naive T cell function by the NF-kappaB2 pathway.. Nat Immunol.

[20] Matsumoto M, Yamada T, Yoshinaga SK, Boone T, Horan T (2002). Essential role of NF-kappa B-inducing kinase in T cell activation through the TCR/CD3 pathway.. J Immunol.

[21] Karrer U, Althage A, Odermatt B, Hengartner H, Zinkernagel RM (2000). Immunodeficiency of alymphoplasia mice (aly/aly) in vivo: structural defect of secondary lymphoid organs and functional B cell defect.. Eur J Immunol.

[22] Gutcher I, Becher B (2007). APC-derived cytokines and T cell polarization in autoimmune inflammation.. J Clin Invest.

[23] Lund FE, Partida-Sanchez S, Lee BO, Kusser KL, Hartson L (2002). Lymphotoxin-alpha-deficient mice make delayed, but effective, T and B cell responses to influenza.. J Immunol.

[24] Moyron-Quiroz JE, Rangel-Moreno J, Hartson L, Kusser K, Tighe MP (2006). Persistence and responsiveness of immunologic memory in the absence of secondary lymphoid organs.. Immunity.

[25] Feuerer M, Beckhove P, Garbi N, Mahnke Y, Limmer A (2003). Bone marrow as a priming site for T-cell responses to blood-borne antigen.. Nat Med.

[26] Moyron-Quiroz JE, Rangel-Moreno J, Kusser K, Hartson L, Sprague F (2004). Role of inducible bronchus associated lymphoid tissue (iBALT) in respiratory immunity.. Nat Med.

[27] Dranoff G, Jaffee E, Lazenby A, Golumbek P, Levitsky H (1993). Vaccination with irradiated tumor cells engineered to secrete murine granulocyte-macrophage colony-stimulating factor stimulates potent, specific, and long-lasting anti-tumor immunity.. Proc Natl Acad Sci U S A.

[28] Junt T, Nakano H, Dumrese T, Kakiuchi T, Odermatt B (2002). Antiviral immune responses in the absence of organized lymphoid T cell zones in plt/plt mice.. J Immunol.

[29] Banks TA, Rouse BT, Kerley MK, Blair PJ, Godfrey VL (1995). Lymphotoxin-alpha-deficient mice. Effects on secondary lymphoid organ development and humoral immune responsiveness.. J Immunol.

[30] Legendre C, Caillat-Zucman S, Samuel D, Morelon S, Bismuth H (1997). Transfer of symptomatic peanut allergy to the recipient of a combined liver-and-kidney transplant.. N Engl J Med.

[31] Klein I, Crispe IN (2006). Complete differentiation of CD8+ T cells activated locally within the transplanted liver.. J Exp Med.

[32] Flajnik MF, Du PL (2004). Evolution of innate and adaptive immunity: can we draw a line?. Trends Immunol.

[33] Stavnezer J, Amemiya CT (2004). Evolution of isotype switching.. Semin Immunol.

[34] Becher B, Durell BG, Miga AV, Hickey WF, Noelle RJ (2001). The clinical course of experimental autoimmune encephalomyelitis and inflammation is controlled by the expression of CD40 within the central nervous system.. J Exp Med.

[35] Greter M, Heppner FL, Lemos MP, Odermatt BM, Goebels N (2005). Dendritic cells permit immune invasion of the CNS in an animal model of multiple sclerosis.. Nat Med.

[36] Gutcher I, Urich E, Wolter K, Prinz M, Becher B (2006). Interleukin 18-independent engagement of interleukin 18 receptor-alpha is required for autoimmune inflammation.. Nat Immunol.

