# Supplementary figures and images for: Neo-Lymphoid Aggregates in the Adult Liver Can Initiate Potent Cell-Mediated Immunity
Source: PLoS Biol. 2009 May 26;7(5):e1000109. doi: 10.1371/journal.pbio.1000109 (PMC2680335; doi:10.1371/journal.pbio.1000109)

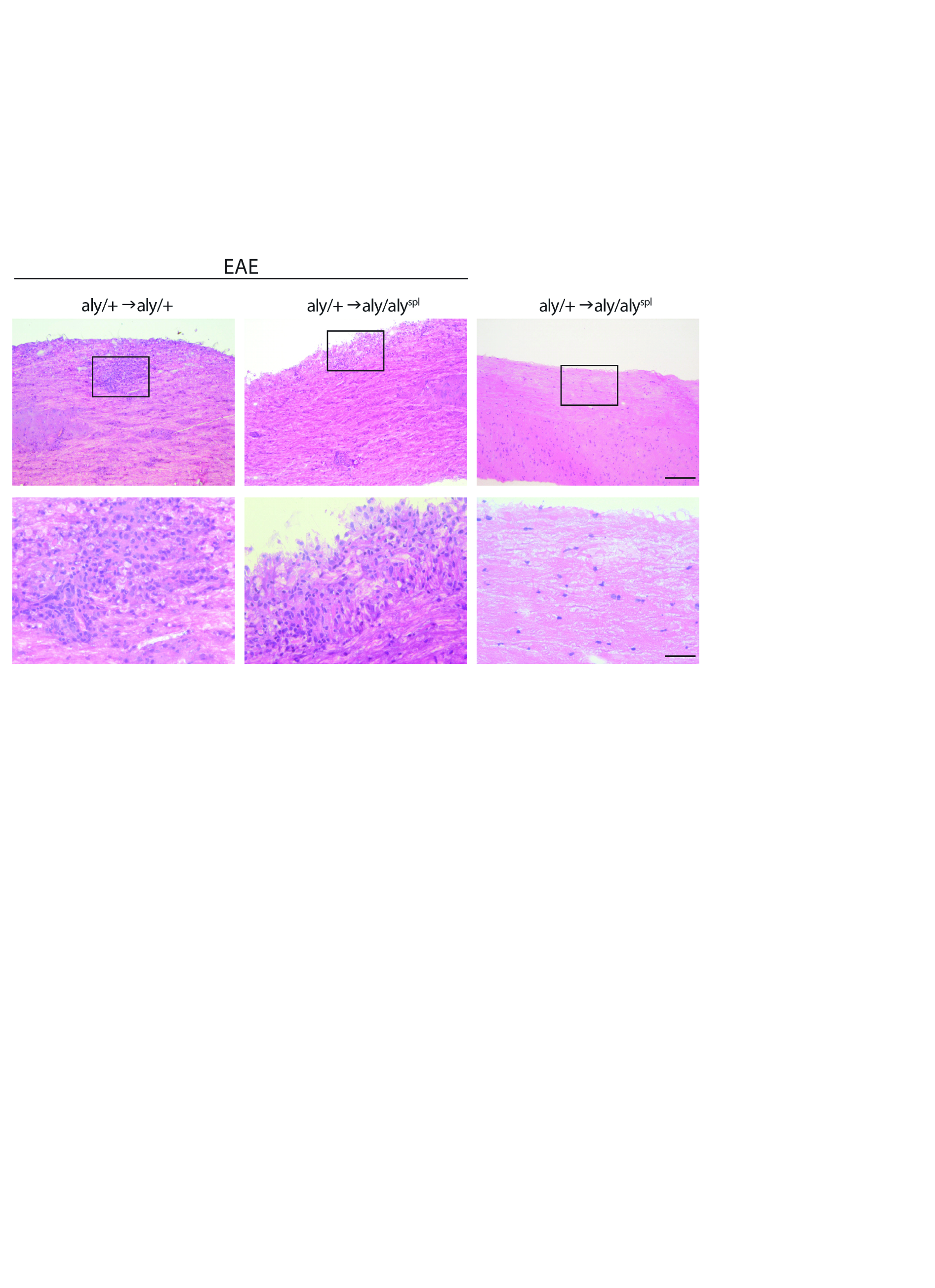

Supplement: Figure S1 — Inflammatory lesions in the CNS of mice lacking SLTs. H&E stainings of spinal cord sections of diseased aly/+→aly/+ and aly/+→aly/aly spl BM-chimeras. Lower row represents higher magnification of the insert in upper row. Bar in upper row indicates 200 µm and in lower row 50 µm. (3.79 MB TIF) [file pbio.1000109.s001.tif]

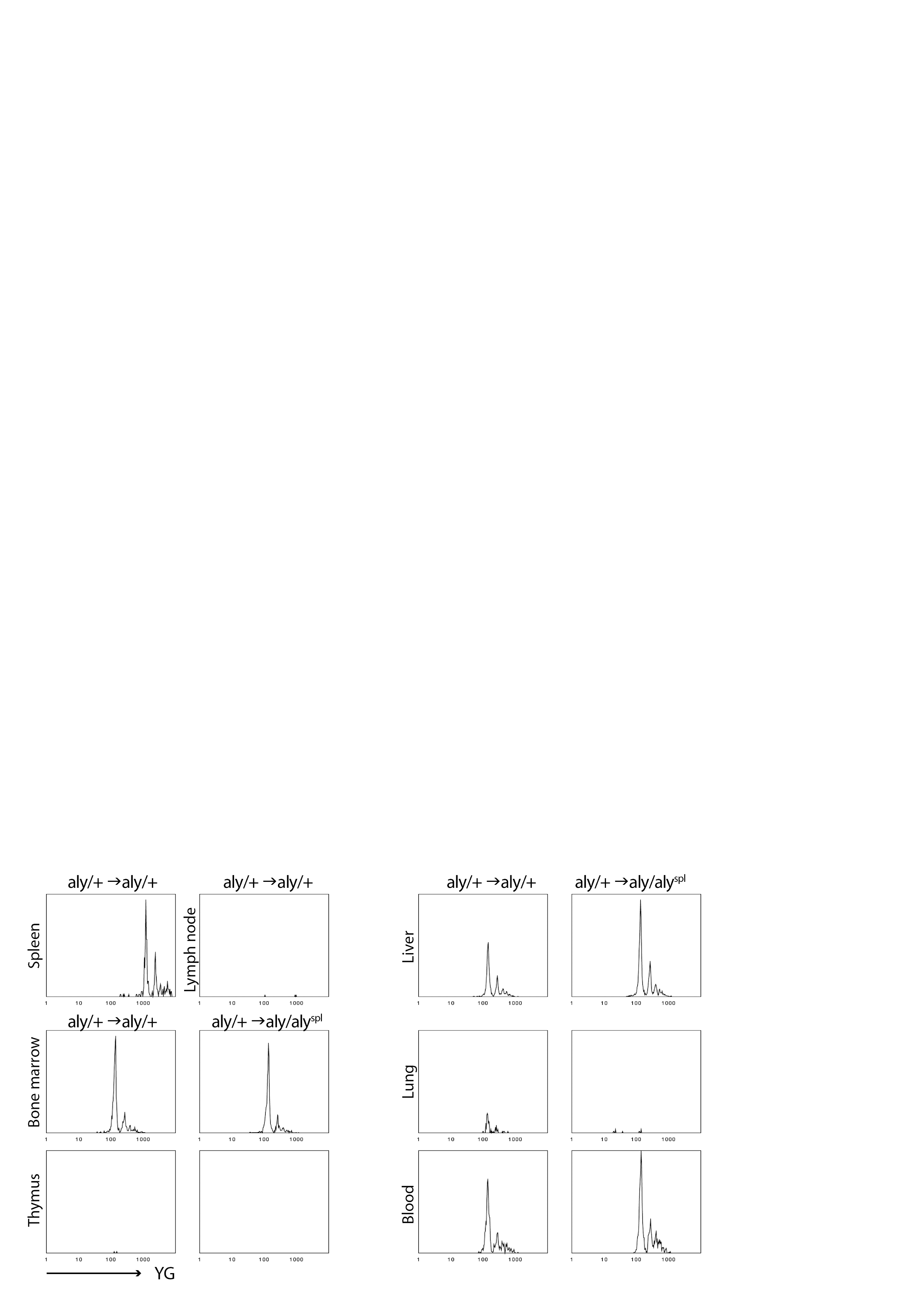

Supplement: Figure S2 — Intravenously delivered Ag accumulates in the spleen, BM, and liver. Aly BM-chimeras were injected i.v. with YG microspheres, and various organs were analyzed by FACS for the presence of fluorescently labeled APCs 7 dpi. Data represent one of three individual experiments. (0.63 MB TIF) [file pbio.1000109.s002.tif]

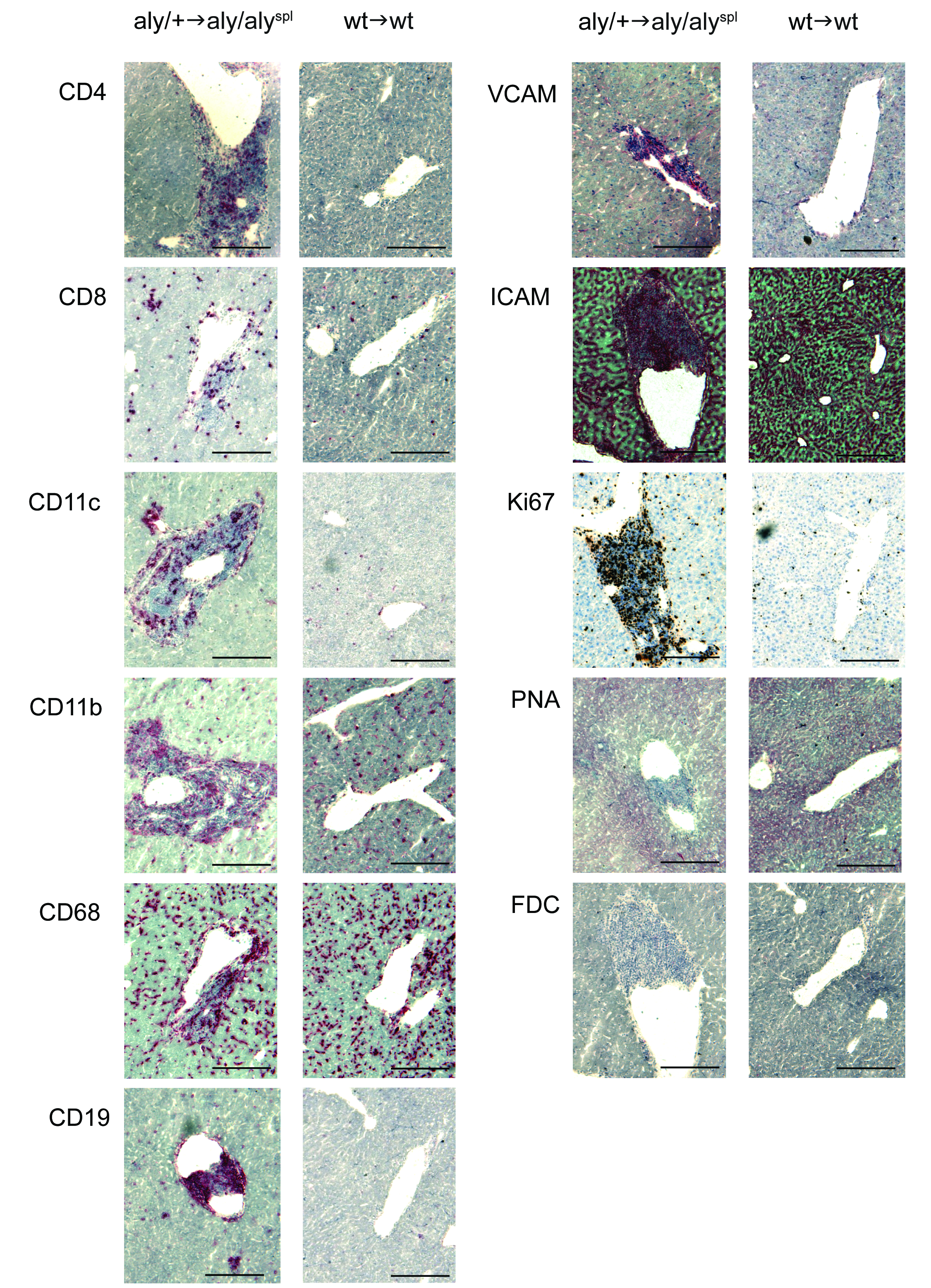

Supplement: Figure S3 — Expression of lymphoid structure markers in livers of aly BM-chimeric mice. Liver cryosections from aly BM-chimeras immunized s.c. with MOG35–55 (d11) were stained with antibodies against CD4, CD8, CD11b, CD11c, CD19, CD62L, CD68, FDC, ICAM, Ki67, PNA, and VCAM. Bar indicates 200 µm. (17.11 MB TIF) [file pbio.1000109.s003.tif]
